# Supplementary material for: Whole-body visualization of SARS-CoV-2 biodistribution in vivo by immunoPET imaging in non-human primates
Source: Nat Commun. 2025 Mar 21;16:2816. doi: 10.1038/s41467-025-58173-y (PMC11928647; doi:10.1038/s41467-025-58173-y)
Supplement: Supplementary file 1 — Supplementary Information [file 41467_2025_58173_MOESM1_ESM.pdf]

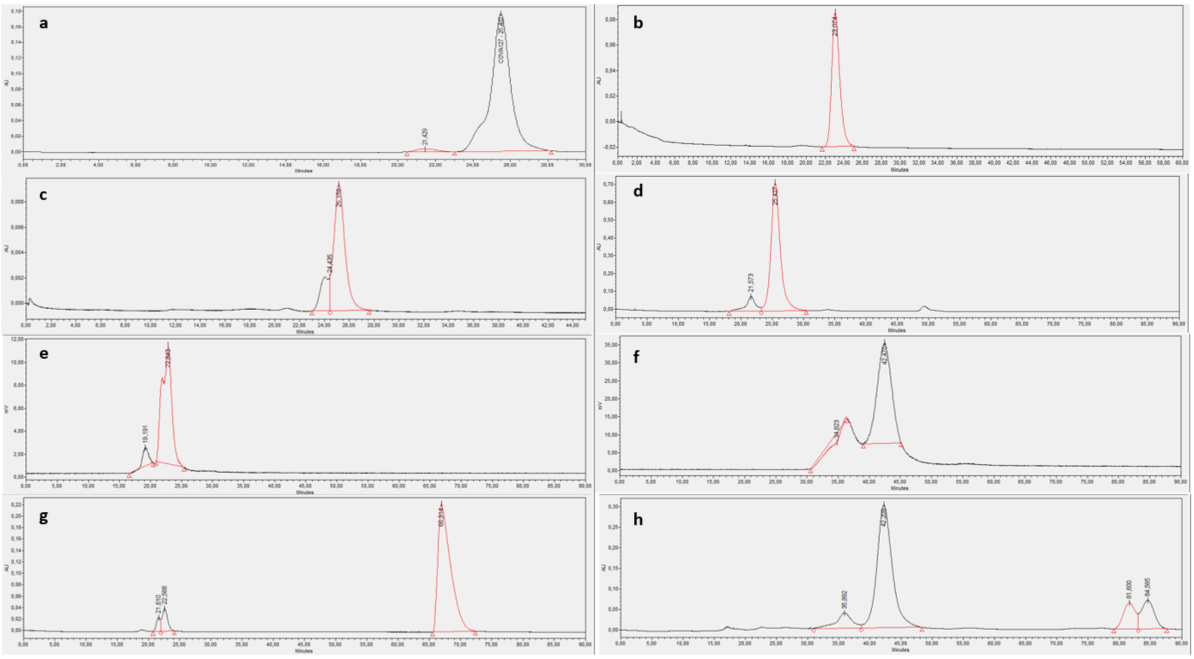

Supplementary figure 1. Size exclusion chromatography chromatograms

(a) SEC chromatogram of COVA 1-27; rt = 25.5 min ; (b) SEC chromatogram of IgG1 kappa; rt = 23.1 min ; (c) SEC chromatogram of COVA 1-27-DFO conjugate; rt = 24.4, 25.2 min ; (d) SEC chromatogram of IgG1 kappa; rt = 23.1 min ; (e) SEC chromatogram of radiolabelled COVA 1-27; γ detection, rt = 19.2, 22.8 min ; (f) SEC chromatogram of radiolabelled IgG1 kappa; γ detection, rt = 34.8, 42.5 min ; g SEC chromatogram of radiolabelled COVA 1-27; UV detection, rt = 21.6, 22.6 min ; (h) SEC chromatogram of radiolabelled IgG1 kappa; UV detection, rt = 35.9, 42.2 min

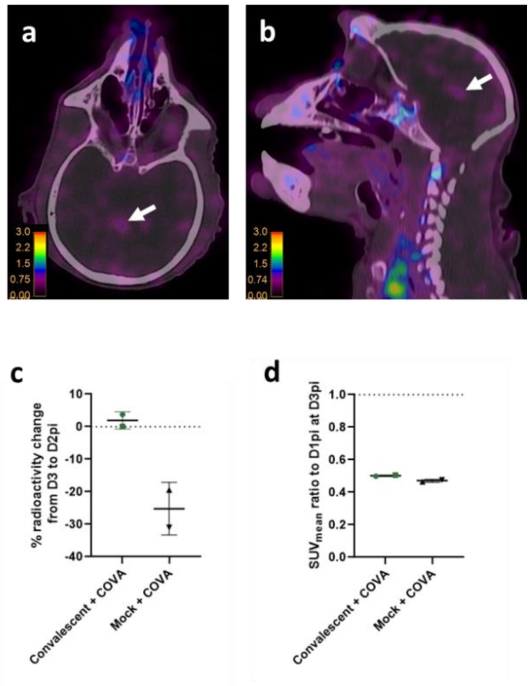

Supplementary figure 2. COVA1-27 uptake in the brain of convalescent animals

(a, b) [^{89}Zr]COVA1-27-DFO PET signal in the brain indicated by the white arrows, sagittal and axial view respectively at D3pi. (CM1). (c, d) Corresponding quantification of the maximum (c) and mean (d) [^{89}Zr]COVA1-27-DFO PET signal in the brain of all animals at D3pi with ratio to D2pi and D1pi respectively. Data are represented as individual values with mean and SD (n = 2 animals per group).

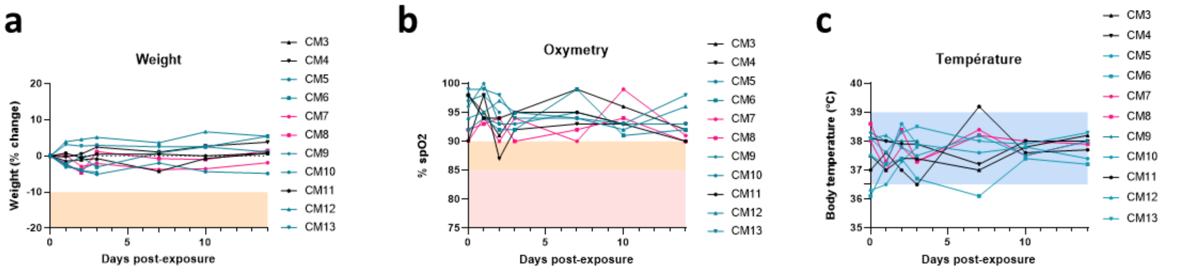

Supplementary figure 3. Clinical observations

(a) Weight, (b) oxymetry and (c) temperature evolution after exposure, mock+COVA group is represented in black (circle, pyramid, triangle), exposed +COVA group is in turquoise (pyramid, triangle, circle, square, diamond, hexagon) and exposed+IgG in pink (circle, square).

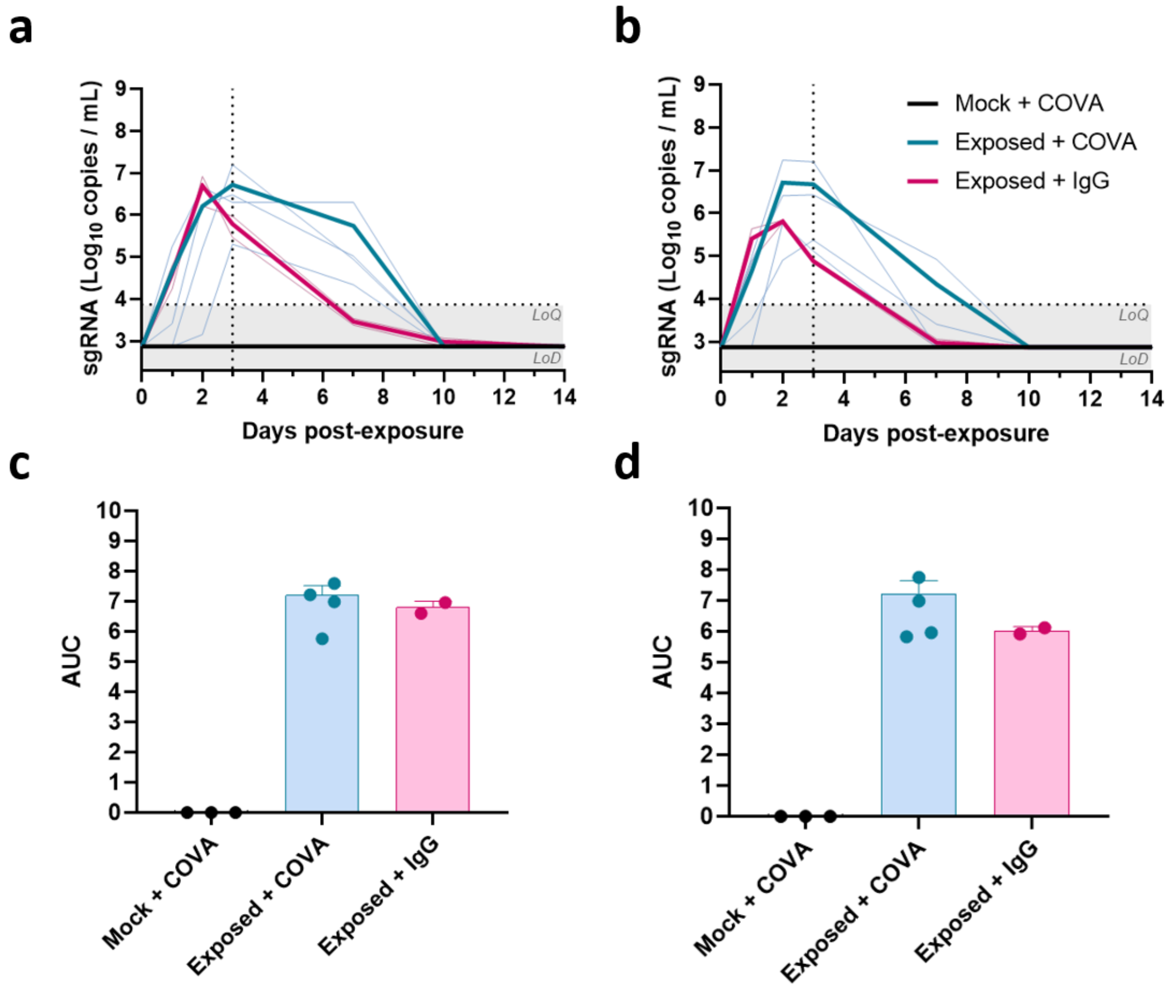

Supplementary figure 4. SARS-CoV-2 subgenomic RNA (sgRNA) titres over time in nasopharynx and trachea
Evolution of the of the SARS-CoV-2 sgRNA titres (Log_{10} copies/mL) over two weeks in the nasopharynx **(**a) and the trachea ; (b) The individual follow up is represented by the thin lines and the average of each group in bold ; Area under the curve (AUC) for each group in the nasopharynx (c) and the trachea (d) mock+COVA group is represented in black (n = 3animals), exposed +COVA group is in turquoise (n = 4 animals) and exposed+IgG in pink (n = 2 animals), individual values are represented by circles

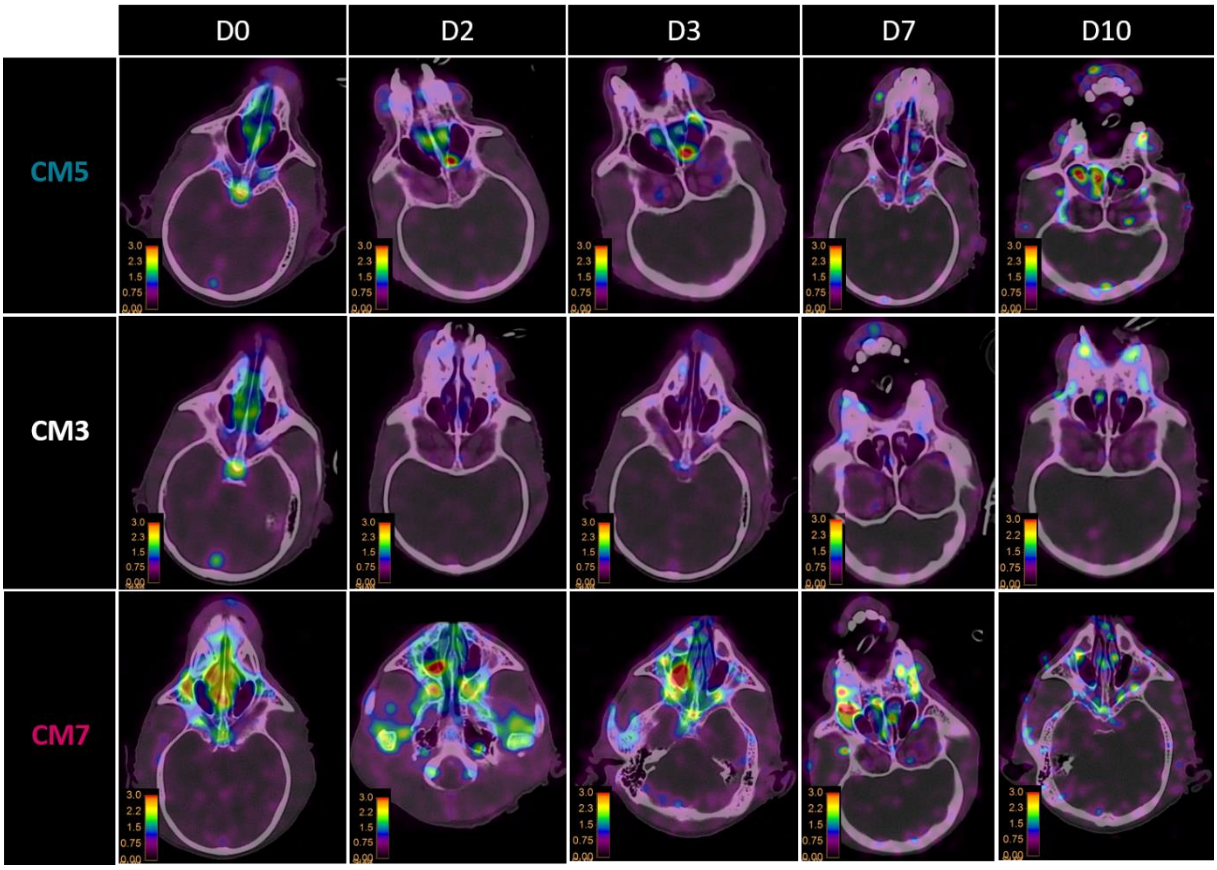

Supplementary figure 5. COVA1-27 uptake in the nasopharynx

Representative images of the nasal cavity at day 0, 2, 3, 7, and 10 post-exposure (pe) for each group: CM5 (turquoise, exposed +COVA), CM3 (white, mock+COVA), and CM7 (pink, exposed+IgG), color scale 0-3 SUV.

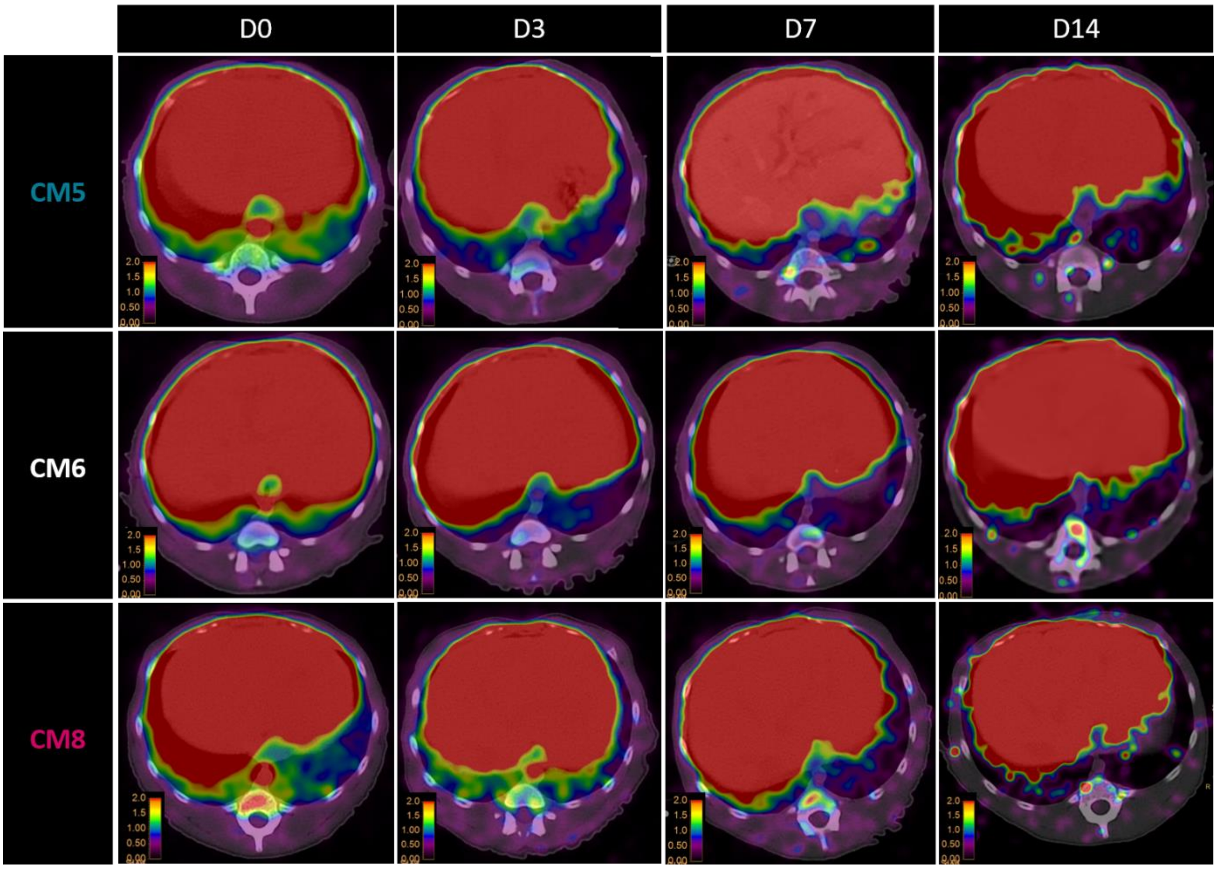

Supplementary figure 6. COVA1-27 uptake in lungs
Representative images of the lungs at day 0, 3, 7, and 14 post-exposure (pe) for each group: CM6 (turquoise, exposed +COVA), CM3 (white, mock+COVA), and CM7 (pink, exposed+IgG), color scale 0-2 SUV.

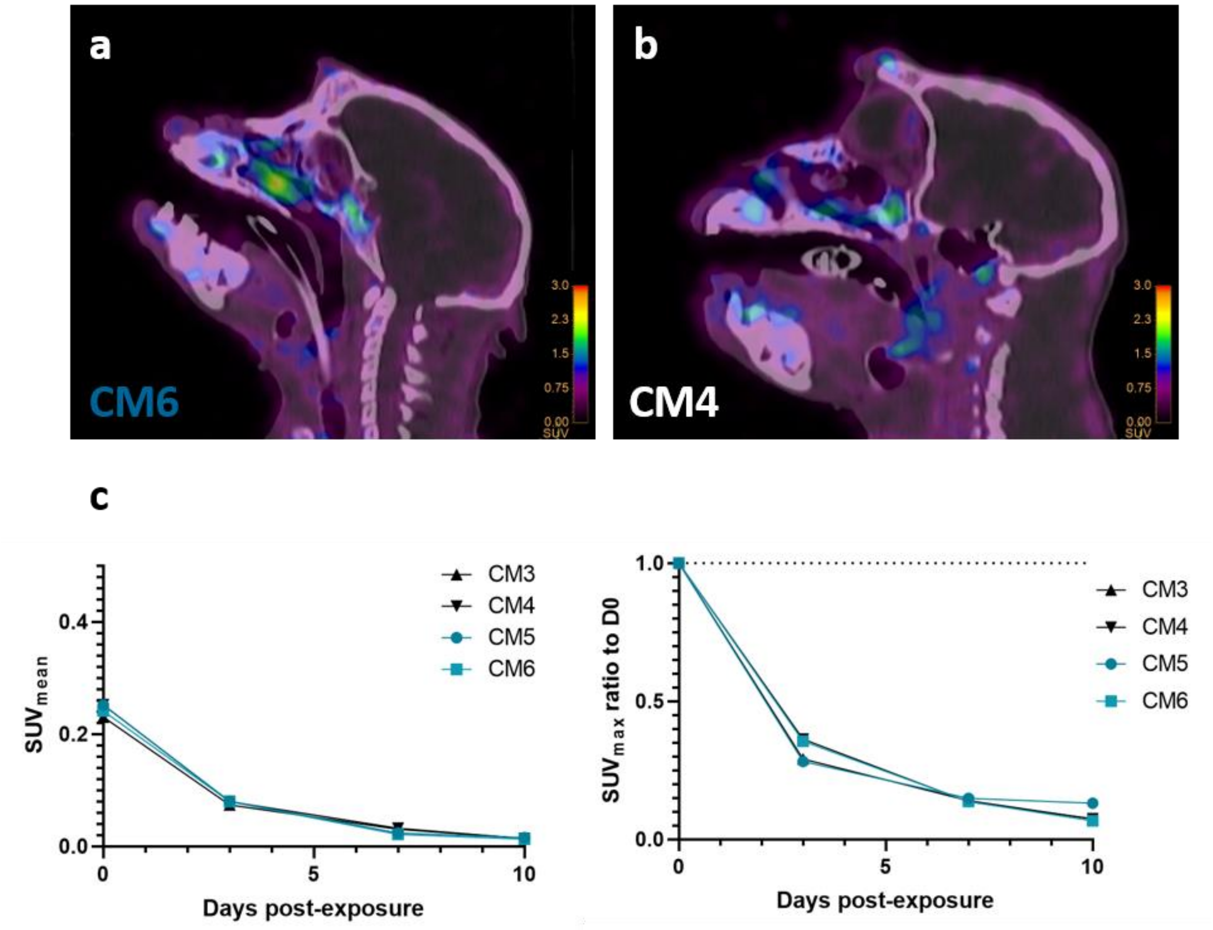

Supplementary figure 7. COVA1-27 uptake assessment in the brain

(a-b) Representative images of the brain at day 3 post-exposure (pe) of animals injected with [^{89}Zr]COVA1-27-DFO for each group: CM6 (turquoise, exposed +COVA), CM4 (white, mock+COVA), color scale 0-3 SUV. (c-d) Average (SUVmean) or maximum (SUVmax) brain [^{89}Zr]COVA1-27-DFO uptake over time in [^{89}Zr]COVA1-27-DFO injected animals exposed (turquoise) or not (black) to SARS-CoV-2

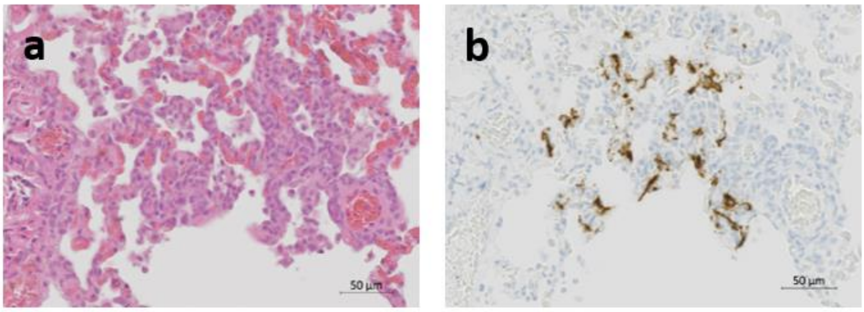

Supplementary figure 8. Representative viral distribution in the lung parenchyma of CM10

(a) Histological characterisation using Hematoxylin Eosin (HE) staining in the caudal right lobe of the lungs of CM10 and corresponding (b) cellular-scale evaluation of the virus distribution (brown signal) using in situ hybridisation (ISH) in the lungs (caudal left lobe)

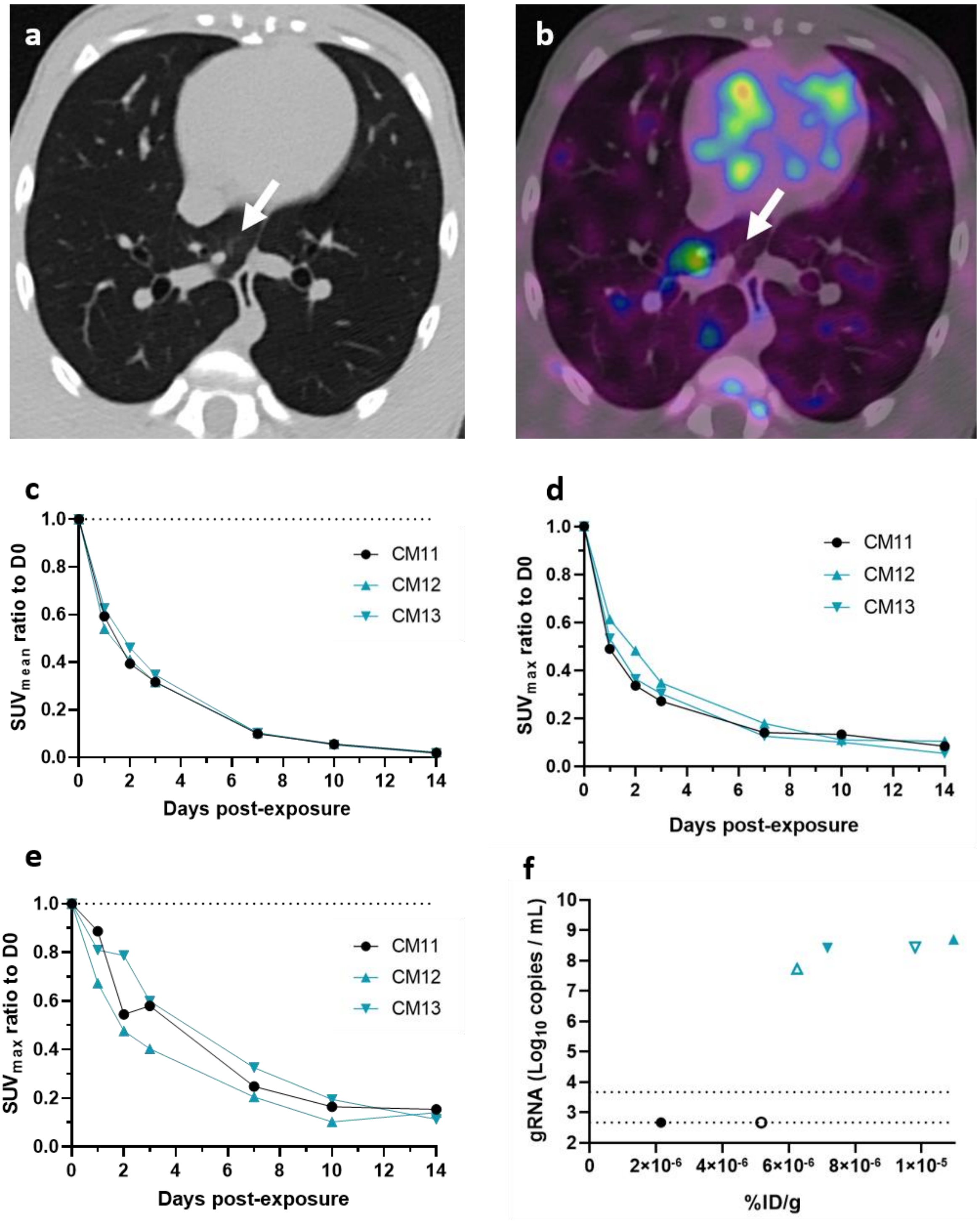

Supplementary figure 9. Low dose COVA1-27 uptake assessment in lungs and nasal cavity
(a) Representative CT lung lesion indicated by the white arrow and (b) its associated PET signal indicated by a white arrow, color scale 0-3 SUV ; (c) Longitudinal evaluation of the normalized PET signal SUV_{mean} and (d) SUV_{max} to D0 post-exposure (0 dpe) in the lungs of animals ; (e) Longitudinal evaluation of the normalized PET signal SUV_{max} to 0 dpe in the nasal cavity of animals ; (f) Evolution from 2 (hollow symbol) to 3 dpe (filled symbol) of the radioactivity in the nasopharyngeal swabs correlated with the SARS-CoV-2 gRNA titres in the same sample

Organ samples list	Included in the analysis
Lung – cranial right lobe	x
Lung – cranial left lobe	x
Lung – caudal right lobe	x
Lung – caudal left lobe	x
Lung –right lingula lobe	x
Lung –left lingula lobe	x
Lung – accessory lobe	x
Trachea	x
Spleen	x
Axillary lymph node	x
Inguinal lymph node	x
Tracheobronchial lymph node	x
Heart	x
Kidney -Right	x
Kidney -Left	x
Liver	

Supplementary table 1. Organ sampling at necropsy

Animal ID	Probe	Tracer dose	Exposition	Group	Follow up time	Nasal rhinorea (Y/N)	Nasal SUVmax ratio at D3	Nasal swab radioactivity at D3 (%ID/g)	Nasal swab RNA titers at D3 (RNA copies/mL)
CM1	COVA1-27	2,5mg	Convalescents SARS-CoV-2 (3months)	Convalescent + COVA	1 week	No	0,44	5,42E-06	0,00E+00
CM2	COVA1-27	2,5mg	Convalescents SARS-CoV-2 (3months)	Convalescent + COVA	1 week	No	0,41	4,62E-05	0,00E+00
CM3	COVA1-27	2,5mg	PBS	Mock + COVA	2 weeks	No	0,45	3,15E-06	0,00E+00
CM4	COVA1-27	2,5mg	PBS	Mock + COVA	2 weeks	No	0,49	1,97E-06	0,00E+00
CM5	COVA1-27	2,5mg	SARS-CoV-2 (B.1.617.2)	Exposed + COVA	2 weeks	Yes	1,00	8,14E-06	2,15E+07
CM6	COVA1-27	2,5mg	SARS-CoV-2 (B.1.617.2)	Exposed + COVA	2 weeks	No	0,51	8,95E-06	2,74E+08
CM7	IgG1	2,5mg	SARS-CoV-2 (B.1.617.2)	Exposed + IgG	2 weeks	Yes	0,63	2,23E-06	1,11E+08
CM8	IgG1	2,5mg	SARS-CoV-2 (B.1.617.2)	Exposed + IgG	2 weeks	No	0,46	4,01E-06	1,52E+08
CM9	COVA1-27	2,5mg	SARS-CoV-2 (B.1.617.2)	Exposed + COVA (eutha D3)	3 days	No	0,47	1,91E-05	/ (sampling issue)
CM10	COVA1-27	2,5mg	SARS-CoV-2 (B.1.617.2)	Exposed + COVA (eutha D3)	3 days	No	0,58	1,08E-05	3,45E+07
CM11	COVA1-27	150µg	PBS	Mock + COVA (low dose)	2 weeks	No	0,58	2,15E-06	0,00E+00
CM12	COVA1-27	150µg	SARS-CoV-2 (B.1.617.2)	Exposed + COVA (low dose)	2 weeks	No	0,40	1,10E-05	5,09E+08
CM13	COVA1-27	150µg	SARS-CoV-2 (B.1.617.2)	Exposed + COVA (low dose)	2 weeks	No	0,60	7,17E-06	2,64E+08

Animal ID	Probe	Tracer dose	Exposition	Group	Follow up time	Lung lesions (Y/N)	Type of lung lesions (number)	Lung lesion ROI SUVmax ratio at D7	Brain SUVmax ratio at D3
CM1	COVA1-27	2,5mg	Convalescents SARS-CoV-2 (3months)	Convalescent + COVA	1 week	No	/	/	0,80
CM2	COVA1-27	2,5mg	Convalescents SARS-CoV-2 (3months)	Convalescent + COVA	1 week	Yes	GGO (n=1)	0,28	0,64
CM3	COVA1-27	2,5mg	PBS	Mock + COVA	2 weeks	No	/	/	0,43
CM4	COVA1-27	2,5mg	PBS	Mock + COVA	2 weeks	No	/	/	0,48
CM5	COVA1-27	2,5mg	SARS-CoV-2 (B.1.617.2)	Exposed + COVA	2 weeks	Yes	GGO (n=3)	0,22	0,50
CM6	COVA1-27	2,5mg	SARS-CoV-2 (B.1.617.2)	Exposed + COVA	2 weeks	Yes	GGO (n=2)	0,34	0,47
CM7	IgG1	2,5mg	SARS-CoV-2 (B.1.617.2)	Exposed + IgG	2 weeks	Yes	GGO (n=3)	0,22	not assessed
CM8	IgG1	2,5mg	SARS-CoV-2 (B.1.617.2)	Exposed + IgG	2 weeks	Yes	GGO (n=1)	0,17	not assessed
CM9	COVA1-27	2,5mg	SARS-CoV-2 (B.1.617.2)	Exposed + COVA (eutha D3)	3 days	Yes	GGO (n=1)	not assessed (eutha D3)	0,46
CM10	COVA1-27	2,5mg	SARS-CoV-2 (B.1.617.2)	Exposed + COVA (eutha D3)	3 days	Yes	GGO (n=2)	not assessed (eutha D3)	0,40
CM11	COVA1-27	150µg	PBS	Mock + COVA (low dose)	2 weeks	No	/	/	not assessed
CM12	COVA1-27	150µg	SARS-CoV-2 (B.1.617.2)	Exposed + COVA (low dose)	2 weeks	Yes	GGO (n=3)	0,18	not assessed
CM13	COVA1-27	150µg	SARS-CoV-2 (B.1.617.2)	Exposed + COVA (low dose)	2 weeks	Yes	GGO (n=2)	0,13	not assessed

Supplementary table 2. Individual data summary
