# Supplementary material for: Whole-body visualization of SARS-CoV-2 biodistribution in vivo by immunoPET imaging in non-human primates
Source: Nat Commun. 2025 Mar 21;16:2816. doi: 10.1038/s41467-025-58173-y (PMC11928647; doi:10.1038/s41467-025-58173-y)
Supplement: Supplementary file 2 — Reporting Summary [file 41467_2025_58173_MOESM2_ESM.pdf]

nature portfolio

Corresponding author(s): Thibaut Naninck

Last updated by author(s): Feb 20, 2025

Reporting Summary

Nature Portfolio wishes to improve the reproducibility of the work that we publish. This form provides structure for consistency and transparency in reporting. For further information on Nature Portfolio policies, see our Editorial Policies and the Editorial Policy Checklist.

Statistics

For all statistical analyses, confirm that the following items are present in the figure legend, table legend, main text, or Methods section.

n/a	Confirmed	
☐	☒	The exact sample size (*n*) for each experimental group/condition, given as a discrete number and unit of measurement
☐	☒	A statement on whether measurements were taken from distinct samples or whether the same sample was measured repeatedly
☐	☒	The statistical test(s) used AND whether they are one- or two-sided<br>*Only common tests should be described solely by name; describe more complex techniques in the Methods section.*
☐	☒	A description of all covariates tested
☐	☒	A description of any assumptions or corrections, such as tests of normality and adjustment for multiple comparisons
☐	☒	A full description of the statistical parameters including central tendency (e.g. means) or other basic estimates (e.g. regression coefficient) AND variation (e.g. standard deviation) or associated estimates of uncertainty (e.g. confidence intervals)
☐	☒	For null hypothesis testing, the test statistic (e.g. *F*, *t*, *r*) with confidence intervals, effect sizes, degrees of freedom and *P* value noted<br>*Give P values as exact values whenever suitable.*
☒	☐	For Bayesian analysis, information on the choice of priors and Markov chain Monte Carlo settings
☒	☐	For hierarchical and complex designs, identification of the appropriate level for tests and full reporting of outcomes
☒	☐	Estimates of effect sizes (e.g. Cohen's *d*, Pearson's *r*), indicating how they were calculated

Our web collection on statistics for biologists contains articles on many of the points above.

Software and code

Policy information about availability of computer code

Data collection	No software was used for data collection.
Data analysis	*Provide a description of all commercial, open source and custom code used to analyse the data in this study, specifying the version used OR state that no software was used.*

For manuscripts utilizing custom algorithms or software that are central to the research but not yet described in published literature, software must be made available to editors and reviewers. We strongly encourage code deposition in a community repository (e.g. GitHub). See the Nature Portfolio guidelines for submitting code & software for further information.

Data

Policy information about availability of data

All manuscripts must include a data availability statement. This statement should provide the following information, where applicable:

- Accession codes, unique identifiers, or web links for publicly available datasets
- A description of any restrictions on data availability
- For clinical datasets or third party data, please ensure that the statement adheres to our policy

All data associated with this study are present in the paper or the Supplementary Materials. The supporting raw data files are available in the Source Data file and deposited in FigShare database (DOI indicated in the manuscript in the Data availability statement)

Research involving human participants, their data, or biological material

Policy information about studies with human participants or human data. See also policy information about sex, gender (identity/presentation), and sexual orientation and race, ethnicity and racism.

Reporting on sex and gender	*Use the terms sex (biological attribute) and gender (shaped by social and cultural circumstances) carefully in order to avoid confusing both terms. Indicate if findings apply to only one sex or gender; describe whether sex and gender were considered in study design; whether sex and/or gender was determined based on self-reporting or assigned and methods used. Provide in the source data disaggregated sex and gender data, where this information has been collected, and if consent has been obtained for sharing of individual-level data; provide overall numbers in this Reporting Summary. Please state if this information has not been collected. Report sex- and gender-based analyses where performed, justify reasons for lack of sex- and gender-based analysis.*
Reporting on race, ethnicity, or other socially relevant groupings	*Please specify the socially constructed or socially relevant categorization variable(s) used in your manuscript and explain why they were used. Please note that such variables should not be used as proxies for other socially constructed/relevant variables (for example, race or ethnicity should not be used as a proxy for socioeconomic status). Provide clear definitions of the relevant terms used, how they were provided (by the participants/respondents, the researchers, or third parties), and the method(s) used to classify people into the different categories (e.g. self-report, census or administrative data, social media data, etc.) Please provide details about how you controlled for confounding variables in your analyses.*
Population characteristics	*Describe the covariate-relevant population characteristics of the human research participants (e.g. age, genotypic information, past and current diagnosis and treatment categories). If you filled out the behavioural & social sciences study design questions and have nothing to add here, write "See above."*
Recruitment	*Describe how participants were recruited. Outline any potential self-selection bias or other biases that may be present and how these are likely to impact results.*
Ethics oversight	*Identify the organization(s) that approved the study protocol.*

Note that full information on the approval of the study protocol must also be provided in the manuscript.

Field-specific reporting

Please select the one below that is the best fit for your research. If you are not sure, read the appropriate sections before making your selection.

☒ Life sciences ☐ Behavioural & social sciences ☐ Ecological, evolutionary & environmental sciences

For a reference copy of the document with all sections, see nature.com/documents/nr-reporting-summary-flat.pdf

Life sciences study design

All studies must disclose on these points even when the disclosure is negative.

Sample size	This study is mainly based on a proof of concept for detecting SARS-CoV-2 by in vivo imaging in NHPs. Groups are therefore of small size limitting statistical analysis of the data obtained. However control groups (non-infected or injected with a non-specific molecule) were introduced to strenghten the data obtained.
Data exclusions	No data was excluded from the analyses.
Replication	Experimental assays performed in vitro were performed in dillution replicates as indicated in the methods section. Quantitative assessment of the viral RNA titers in the samples were also performed in duplicates. However, data obtained in vivo by imaging could not be so far replicated in an other animal cohort but imaging findings were supported by tissue or fluid analysis from these animals.
Randomization	Cynomolgus macaques were randomly divided into the different groups.
Blinding	Formal blinding of investigators was not included for the majority of the analyses as this study was based on a proof of concept. However, quantitative measurement of viral RNA titers as well as gamma counting in all samples were blind from the experimentators to avoid bias in correlations between imaging findings and samples data.

Reporting for specific materials, systems and methods

We require information from authors about some types of materials, experimental systems and methods used in many studies. Here, indicate whether each material, system or method listed is relevant to your study. If you are not sure if a list item applies to your research, read the appropriate section before selecting a response.

Materials & experimental systems

n/a	Involved in the study	
☐	☒	Antibodies
☐	☒	Eukaryotic cell lines
☒	☐	Palaeontology and archaeology
☐	☒	Animals and other organisms
☒	☐	Clinical data
☒	☐	Dual use research of concern
☒	☐	Plants

Methods

n/a	Involved in the study	
☒	☐	ChIP-seq
☒	☐	Flow cytometry
☒	☐	MRI-based neuroimaging

Antibodies

Antibodies used	COVA1-27 human IgG1 (Provided by Rogier W. Sander's lab, as described in the method section)<br>Human IgG isotype control (Invitrogen catalog n° 31154)
Validation	COVA1-27 validation was done in our experiment . Validation statements and data for the IgG isotype control antibody can be found on the manufacturers' website

Eukaryotic cell lines

Policy information about cell lines and Sex and Gender in Research

Cell line source(s)	HEK293F (Invitrogen cat n°R 79007)
Authentication	Authentication for all cell lines was supplied by the commercial supplier, InVivoGen.<br>No further authentication was performed.
Mycoplasma contamination	All cell lines were guaranteed mycoplamsa-free by the supplier
Commonly misidentified lines (See ICLAC register)	*Name any commonly misidentified cell lines used in the study and provide a rationale for their use.*

Animals and other research organisms

Policy information about studies involving animals; ARRIVE guidelines recommended for reporting animal research, and Sex and Gender in Research

Laboratory animals	NHP - Cynomolgus macaques (Macaca fascicularis), aged 3.19 ± 0.47 years and originating from Mauritian AAALAC certified breeding centers, were used in this study
Wild animals	N/A
Reporting on sex	Both male (n=6) and female (n=7) NHPs were used in these studies. We tried to gender balance each group.
Field-collected samples	N/A
Ethics oversight	Young adult male and female CMs (Macaca fascicularis, 6 males and 7 females, aged 3.19 ± 0.47 years), F1 and F2 generations originating from Mauritian AAALAC-certified breeding centers, were used in this study. All animals were exempt from previous SARS-CoV-2 infection and did not exhibit lung lesions as observed by CT before the beginning of this study.<br>All CMs were housed at the IDMIT infrastructure facilities (CEA, Fontenay-aux-roses) under BSL-2 and BSL-3 containment, (Animal facility authorization #D92-032-02, Préfecture des Hauts de Seine, France) and in compliance with European Directive 2010/63/EU, French regulations, and the Standards for Humane Care and Use of Laboratory Animals of the Office for Laboratory Animal Welfare (OLAW, assurance number #A5826-01, US). The protocols were approved by the institutional ethical committee 'Comité d'Ethique en Expérimentation Animale du Commissariat à l'Energie Atomique et aux Energies Alternatives' (CEtEA number 44) under statement number A20-063. The study was authorized by the ''Research, Innovation and Education Ministry'' under registration number APAFIS #28752-2020121710032163v1.

Note that full information on the approval of the study protocol must also be provided in the manuscript.

Plants

Seed stocks	*Report on the source of all seed stocks or other plant material used. If applicable, state the seed stock centre and catalogue number. If plant specimens were collected from the field, describe the collection location, date and sampling procedures.*
Novel plant genotypes	*Describe the methods by which all novel plant genotypes were produced. This includes those generated by transgenic approaches, gene editing, chemical/radiation-based mutagenesis and hybridization. For transgenic lines, describe the transformation method, the number of independent lines analyzed and the generation upon which experiments were performed. For gene-edited lines, describe the editor used, the endogenous sequence targeted for editing, the targeting guide RNA sequence (if applicable) and how the editor was applied.*
Authentication	*Describe any authentication procedures for each seed stock used or novel genotype generated. Describe any experiments used to assess the effect of a mutation and, where applicable, how potential secondary effects (e.g. second site T-DNA insertions, mosiacism, off-target gene editing) were examined.*
